# A simple intravenous glucose tolerance test for assessment of insulin sensitivity

**DOI:** 10.1186/1742-4682-8-12

**Published:** 2011-05-02

**Authors:** Robert G Hahn, Stefan Ljunggren, Filip Larsen, Thomas Nyström

**Affiliations:** 1 Section for Anesthesia, Faculty of Health Sciences, Linköping University, Linköping, Sweden; 2 Research Unit, Södertälje Hospital, Södertälje, Sweden; 3 Karolinska Institutet, Department of Clinical Science and Education, Södersjukhuset, Section of Internal Medicine, Södersjukhuset, Sweden; 4 Karolinska institutet, Department of Physiology and Pharmacology, Stockholm, Sweden

## Abstract

**Background:**

The aim of the study was to find a simple intravenous glucose tolerance test (IVGTT) that can be used to estimate insulin sensitivity.

**Methods:**

In 20 healthy volunteers aged between 18 and 51 years (mean, 28) comparisons were made between kinetic parameters derived from a 12-sample, 75-min IVGTT and the M_bw _(glucose uptake) obtained during a hyperinsulinemic euglycemic glucose clamp. Plasma glucose was used to calculate the volume of distribution (*V*_d_) and the clearance (*CL*) of the injected glucose bolus. The plasma insulin response was quantified by the area under the curve (AUC_ins_). Uptake of glucose during the clamp was corrected for body weight (M_bw_).

**Results:**

There was a 7-fold variation in M_bw_. Algorithms based on the slope of the glucose-elimination curve (*CL/V*_d_) in combination with AUC_ins _obtained during the IVGTT showed statistically significant correlations with M_bw_, the linearity being r^2 ^= 0.63-0.83. The best algorithms were associated with a 25-75^th ^prediction error ranging from -10% to +10%. Sampling could be shortened to 30-40 min without loss of linearity or precision.

**Conclusion:**

Simple measures of glucose and insulin kinetics during an IVGTT can predict between 2/3 and 4/5 of the insulin sensitivity.

## Introduction

The best established methods of measuring insulin resistance are the hyperinsulinemic euglycemic glucose clamp and the intravenous glucose tolerance test (IVGTT), of which former is the "gold standard" [1-3]. These methods have a long history as investigative tools in diabetes research but are too cumbersome to be used during surgery, although insulin resistance develops in this setting [4,5].

The aim of this project is to evaluate a simplified IVGTT test that lasts for 30, 40 or 75 min. This test is less labour-intensive than both the glucose clamp and the conventional IVGTT. Analysis of the data is based on a comparison between the "strength" of the insulin response and the elimination kinetics of glucose. A commonly used expression for the "strength" of a physiological factor is the area under the curve (AUC), which was applied here on insulin, while the slope of the elimination curve for glucose served to quantify the "effect".

The hypothesis was that the test could predict insulin resistance with the same or higher precision than the "minimal model" (MINMOD) which is typically based on a longer IVGTT and quite demanding mathematically [6,7]. We assessed this objective by comparing the simplified IVGTT with the result of the glucose clamp in 20 healthy volunteers.

## Materials and methods

Twenty non-obese healthy volunteers, 8 females and 12 males, aged between 18 and 51 (mean, 28) years and with a body weight of 49-88 (mean, 68) kg, were studied. None of them had any disease requiring medication, and routine blood chemistry confirmed the absence of metabolic disease (Table 1, top). The study was approved by the Regional Ethics Committee in Stockholm and complied with the Helsinki Declaration. Each volunteer gave his/her written consent to participate.

**Table 1:** Baseline data and key results for the IVGTT and the glucose clamp.

Parameter	Mean (SD), or median (25^th^-75^th ^percentiles)	Unit
**Health status**		
Body mass index	23.4 (2.3)	kg/m^2^
HbA1c	44 (0.5)	mmol/mol
Blood Hb concentration	126 (14)	mmol/L;
Serum creatinine concentration	83 (3)	μmol/L
Serum sodium and potassium concentrations	141 (2); 3.9 (0.3)	mmol/L
**IVGTT**		
Plasma glucose, baseline	4.8 (0.5)	mmol L^-1^
Plasma insulin, baseline	21 (12-24)	pmol L^-1^
Volume of distribution (*V*_d_)	14.0 (6.5)	L
per kg body weight	0.20 (0.09)	L kg^-1^
Clearance (*CL*)	0.63 (0.26)	L min^-1^
per kilo body weight	9.3 (3.8)	ml min^-1 ^kg^-1^
Insulin sensitivity (S_I_) of MINMOD	16 (7-32)	10^-5 ^L pmol^-1 ^min^-1^
Glucose effectiveness (S_G_) in MINMOD	13 (5-26)	10^-3 ^min^-1^
**Glucose clamp**		
Plasma glucose, baseline	5.0 (1.0)	mmol L^-1^
Plasma insulin, baseline	16 (7-30)	pmol L^-1^
Plasma glucose, mean 90-120 min	5.7 (0.3)	mmol L^-1^
Plasma insulin, mean 90-120 min	167 (34)	pmol L^-1^
Glucose metabolism, M, 90-120 min	3.1 (1.2)	mmol min^-1^
M_bw _= per kg body weight	45 (15)	μmol min^-1 ^kg^-1^

IVGTT = intravenous glucose tolerance test

### Euglycemic hyperinsulinemic clamp

The subjects reported at the laboratory between 7.30-8.00 AM. A superficial dorsal hand vein was cannulated in retrograde direction with a small three-way needle and kept patent by repeated flushing with saline solution. The hand and lower arm were warmed by a heating pad for intermittent sampling of arterialized venous blood for glucose determination (Hemocue, Ängelholm, Sweden). In the opposite arm an intravenous catheter was inserted into the left antecubital vein for insulin and glucose infusion.

During the 120-min test, insulin 20 mU · BSA m^-2 ^· min^-1 ^(Human Actrapid, NovoNordisk A/S, Bagsverd, Denmark) was infused along with 20% dextrose (Fresenius Kabi, Uppsala, Sweden). Baseline blood samples were drawn and the euglycemic hyperinsulinemic clamp was initiated by infusion of a bolus dose of insulin for 4 minutes followed by a step-wise increase in glucose for 10 min. The glucose infusion rate was adjusted to keep the subjects' blood glucose level constant at 5 mmol/L on the basis of arterialized samples withdrawn every 5 min from the dorsal hand vein catheter [8]. The infusion rate during the last 30 min, after correction for body weight, was taken to represent the metabolism of glucose (*M*_bw_) [1-3].

### Intravenous glucose tolerance test

On the second occasion, 1-2 days apart from the clamp study and after 12 h of fasting, a regular intravenous glucose tolerance test (IVGTT) was performed to determine the early insulin response phase (0-10 min), as well as the area-under-the-curve for insulin (AUC*_ins _*being total insulin and ΔAUC*_ins _*above baseline) and C-peptide for up to 75 minutes. A bolus of glucose (300 mg/kg in a 30% solution) was given within 60 sec into the antecubital vein. Blood was sampled from the contralateral antecubital vein at 0, 2, 4, 6, 8, 10, 20, 30, 40, 50, 60 and 75 min for assessment of the plasma glucose, insulin, and C-peptide concentrations. Plasma glucose was measured by the glucose oxidase method used by the hospital's routine laboratory. Plasma insulin and C-peptide were measured using ELISA kits (Mercodia AB, Uppsala, Sweden).

### Calculations

The pharmacokinetics of the glucose load was analysed using a one-compartment open model [9]. Here, the plasma concentration (*G*) at any time (t) resulting from infusing glucose at the rate *R*_o _is calculated from the following differential equation:

where *G_b _*is the baseline glucose, *V*_d _is the volume of distribution, *CL *the clearance and *CL*/*V*_d _the slope of the glucose elimination curve. The half-life (T_1/2_) of the exogenous glucose load was obtained as (ln 2 *V*_d _/*CL*). The AUC for plasma insulin was calculated by using the linear trapezoid method.

The glucose and insulin data were also analyzed by applying the "minimal model" (MINMOD) of Bergman *et al*. [6,7]. The kinetic system consists of two differential equations:

where *S*_I _= glucose sensitivity, *S*_G _= glucose effectiveness, *X*(t) is insulin action in the interstitial fluid space, and *F*(t) a function for the elevation of plasma insulin above the basal level. *p*_2 _is the removal rate of insulin from the interstitial fluid space while *p*_3 _describes the movement of circulating insulin to the interstitial space.

The best estimates for the unknown parameters in these models were estimated for each of the 20 experiments individually by nonlinear least-squares regression. No weights were used. The mathematical software was Matlab R2010a (MathWorks, Natick, MA, USA).

The insulin sensitivity was also quantified by "Quicki", which is the inverse of the logarithm of the product of plasma glucose and plasma insulin at baseline [10]. Finally, we tested the recently proposed equation by Tura *et al*. [11] for short IVGTTs:

where *CS*_1 _a surrogate measure for insulin sensitivity, *K*_G _is the slope of the glucose elimination curve (same as *CL*/*V*_d_) and *T *is the time after 10 min.

### Statistics

The results were presented as mean and standard deviation (SD) and, when there was a skewed distribution, as the median (25^th^-75^th ^percentile range). Simple or multiple linear regression analysis, in which r^2 ^is the coefficient of determination, was used to express "linearity" when studying the relationship between the M_bw _of the glucose clamp (control) and various algorithms for insulin sensitivity derived from data collected during the IVGTT. The error in the prediction of M_bw _associated with each regression analysis was obtained as [100% (fitted-measured)/measured]. The change in prediction error obtained by restricting the analysis period from 75 to 40 and 30 min was tested by Friedman's test. All reported correlations were statistically significant by *P *< 0.05.

## Results

### Clamp

M_bw _of the glucose clamp varied 7-fold (Table 1, middle). Between 2/3 and 4/5 of this variability could be predicted by linear regression based on indices of glucose and insulin turnover obtained from the data collected during the IVGTT.

### IVGTT

**Figure 1:**
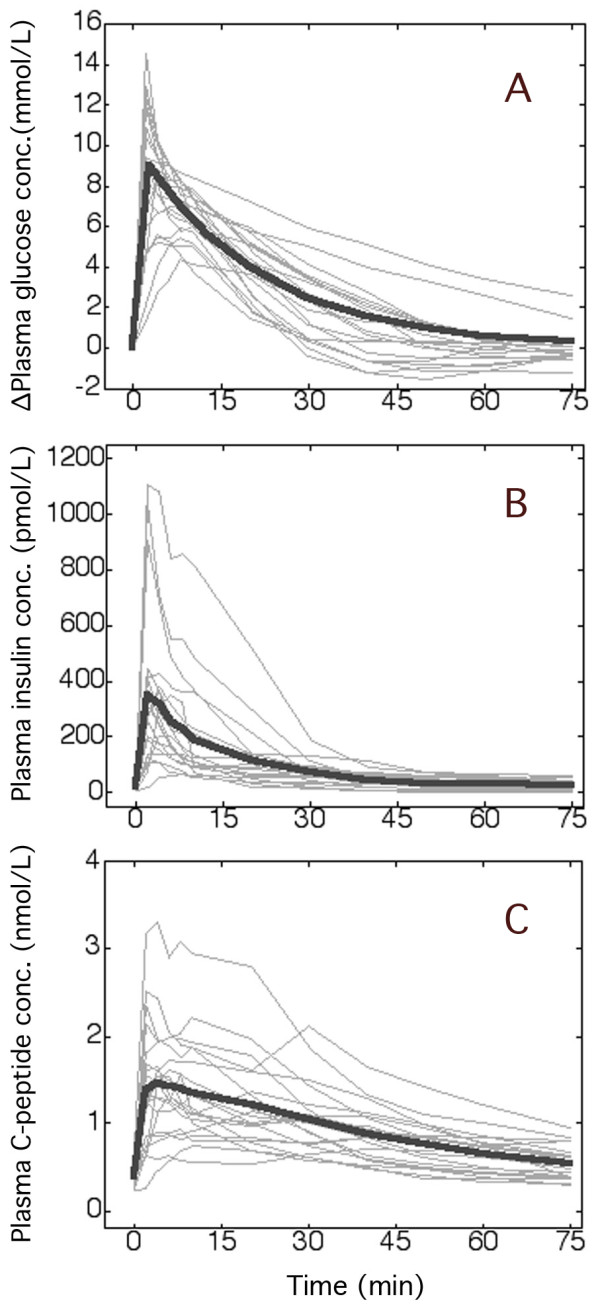
**Plasma concentrations during the IVGTT**. Plasma glucose above baseline (**A**) and the plasma insulin (**B**) and C-peptide concentrations (**C**) during 20 intravenous glucose tolerance tests (IVGTTs). The thin lines represent one experiment. The thick line in A is the modelled average curve, based on the kinetic data shown in Table 1, while B and C are the mean for each point in time.

All 20 experiments could be analysed with the proposed equations for plasma glucose and insulin kinetics (Figure 1; Table 1, bottom). However, the glucose kinetics of 3 experiments were studied only up to 40 min due to rapid elimination followed by mild hypoglycemia, which otherwise distorted the elimination slope.

### First key algorithm

**Figure 2:**
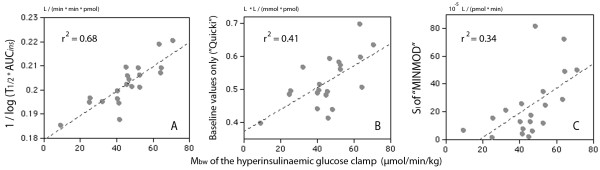
**Insulin resistance as given by the glucose clamp and a short IVGTT**. (**A**) The relationship between M_bw _of the hyperinsulinemic euglycemic clamp and a surrogate expression for insulin sensitivity based on the half-life of glucose and the area under the curve (AUC) for plasma insulin during a 75-min IVGTT in 20 volunteers. (**B**) Same equation but using only baseline plasma glucose and insulin concentrations. (**C**) M_bw _versus insulin sensitivity obtained by "minimal model" (MINMOD) analysis.

One useful algorithm contained the ^10^log of the product of T_1/2 _for the exogenous glucose load and AUC for plasma insulin. Various modifications of the algorithm correlated with M_bw _with a linearity of r^2 ^= 0.63-0.68 (Figure 2A, Table 2).

**Table 2:** Linear correlations between the IVGTT and the glucose clamp.

Y	X	Equation	Time period	r^2^	25^th-^75^th ^percentiles of prediction error	
M_bw_		Y = -172 + 1040 X	75 min	0.63	-10%	+16%
		Y = -201 + 1179 X	40 min	0.63	-8%	+20%
		Y = -219 + 1256 X	30 min	0.62	-12%	+26%
	Same equation, but using total insulin AUC	Y = -220 + 1310 X	75 min	0.68	-11%	+9%
		Y = -218 + 1287 X	40 min	0.63	-8%	+12%
		Y = -248 + 1419 X	30 min	0.66	-8%	+20%
M_bw_		Y = -19 +124 X	Baseline "Quicki"	0.41	-14%	+11%
M_bw_	*S*_I _of MINMOD 10^-5^	Y = 36 + 0.38 X	75 min	0.34	-16%	+24%

Equations compare the cellular uptake of glucose obtained by the glucose clamp (M_bw,; _μmol min^-1 ^kg^-1^) and indices of glucose kinetics and plasma insulin obtained during an intravenous glucose tolerance test (IVGTT) in 20 non-obese volunteers.

*T*_1/2 _= half-life of exogenous glucose (units: min)

Glucose_o_, Ins_o _= plasma concentrations of glucose and insulin at baseline (units: mmol L^-1 ^and pmol L^-1^)

AUC*_ins _*= area under the curve for plasma insulin over time (unit: pmol min L^-1^)

MINMOD = "minimal model analysis" according to Bergman *et al*. [6]

Consistently weaker correlations were obtained on correcting M_bw _for the steady state plasma glucose and insulin concentrations (data not shown, r^2^≈0.40-0.50).

This key algorithm has the same construction as "Quicki" which uses only the baseline values of plasma glucose and insulin. The original "Quicki" equation correlated with M_bw _with a linearity of only r^2 ^= 0.41 (Figure 2B) which was still slightly stronger than for other similar expressions, such as HOMA-IR (r^2 ^= 0.35) and the G/I ratio (r^2 ^= 0.39) [2].

### MINMOD and Tura's equation

Weaker correlations were also obtained when comparing M_bw _with the insulin sensitivity as obtained by "minimal model analysis" (MINMOD) of the IVGTT data (r^2 ^= 0.34, Figure 2C). Plots of *X*(t) obtained by MINMOD indicated that the insulin concentration at the effect site was highest at 18 min (13-33) min.

The recently published equation by Tura *et al*. [11] correlated with M_bw _with a linearity of r^2 ^= 0.54 for the period 0-40 min. Logarithm-transformation of Tura's surrogate measure for insulin sensitivity increased r^2 ^to 0.65.

### Second key algorithm

**Figure 3:**
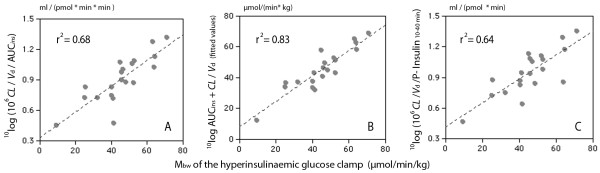
**Insulin resistance by the glucose clamp and a short IVGTT**.  The relationship between M_bw _and various combinations of the clearance (*CL*) and volume of distribution (*V*_d_) of glucose and (**A, B**) the area under the curve for plasma insulin (AUC_ins_) during the 75-min IVGTT, or (**C**) using the mean plasma insulin level measured at 10, 20, 30, and 40 min.

Another equation applied the parameters of the glucose kinetics directly and might therefore be easier to handle (Table 3, Figure 3A).

**Table 3:** Further linear correlations between the IVGTT and the glucose clamp.

Y	X	Equation	Time period	r^2^	25^th-^75^th ^percentiles of prediction error	
M_bw_		Y = -2.5 + 45.4 X	75 min	0.64	-10%	+16%
		Y = -8.6 + 51.5 X	40 min	0.64	-8%	+21%
		Y = -13.8 + 54.9 X	30 min	0.64	-12%	+25%
	Same equation, but using total insulin AUC	Y = -2.8 + 53.4 X	75 min	0.68	-10%	+9%
		Y = -6.1 + 54.0 X	40 min	0.64	-8%	+13%
		Y = -14.5 + 60.0 X	30 min	0.67	-8%	+20%
M_bw_		Y = 206 - 49.0 X + 340 *CL/V_d_*	75 min	0.70	-11%	+16%
		Y = 224 - 56.4 X + 480 *CL/V*_d_	40 min	0.74	-10%	+20%
		Y = 223 - 57.9 X + 580 *CL/V_d_*	30 min	0.70	-10%	+23%
	Same equation, but using total insulin AUC	Y = 265 - 63.6 X + 383 *CL/V_d_*	75 min	0.83	-9%	+11%
		Y = 262 - 65.4 X + 488 *CL/V_d_*	40 min	0.82	-10%	+11%
		Y = 260 - 67.1 X + 602 *CL/V_d_*	30 min	0.79	-8%	+14%
M_bw_		Y = -99 + 54.0 X	75 min	0.63	-10%	+16%
		Y = -9 + 51.5 X	10-40 min	0.64	-8%	+21%
		Y = -14 + 54.9 X	10-30 min	0.64	-12%	+26%

*V*_d_, *CL *= volume of distribution and clearance of glucose for the IVGTT (units: L and L min^-1^, respectively).

Ins_*mean*, _= mean value plasma of insulin (units: pmol L^-1^)

AUC*_ins _*= area under the curve for plasma insulin over time (unit: pmol min L^-1^)

A promising modification of this second key algorithm inserted the parameters of the glucose kinetics and the AUC for plasma insulin in a multiple regression equation, which yielded a maximum linearity of r^2 ^= 0.83 for the relationship between the IVGTT and M_bw _(Table 3, Figure 3B).

Slight strengthening of the linearity was always obtained by using AUC*_ins _*without correction for the baseline plasma insulin level (Tables 2 and 3).

### Exploratory analyses

Replacing AUC*_ins _*by the sum of the plasma insulin concentrations for various periods of time did not greatly impair linearity or the prediction error (Table 3, Figure 3C).

The overall linear correlation between the AUC for C-peptide and insulin was r^2 ^= 0.66. However, replacing AUC_ins _by AUC for C-peptide in the equations proposed above greatly reduce their linearity with M_bw _(r^2 ^≈ 0.20).

## Discussion

### IVGTT *versus *the glucose clamp

The present study searched for an approach to estimate insulin sensitivity that requires only minimum of resources. The results are presented as a number of regression equations that compare M_bw _of the glucose clamp (control) with minor mathematical variations of two key algorithms based on data derived from a short IVGTT. Any of them may be used as substitutes for a glucose clamp in healthy volunteers, although some offer stronger linearity and a smaller prediction error than others.

The first of the key algorithms, shown on top of Table 2, is constructed in a way similar to the "Quicki" [10]. However, the linearity was much stronger when based on the IVGTT as compared to the baseline data used in the "Quicki" (Figure 2A, B).

Various modifications of the second key algorithm, presented in Table 3, were also tested. A promising change was to consider the sum of the slope of the glucose elimination curve, *CL*/*V*_d_, and the insulin "pressure", AUC*_ins_*, in a multiple regression equation. This approach could explain up to 83% of the inter-individual variability in M_bw _(Figure 3).

Reducing the sampling time from 75 min to 40 min, or even 30 min, had only small undue effects on our quality measures, i.e. the linearity and the prediction error.

### Corrections for baseline concentrations

The relationship between plasma insulin and glucose is not a simple one. The dose-response curve is hyperbolic (saturation kinetics) [2,3] and the *CL *of glucose is related to the ^10^log of the insulin level [3,12].

The saturation kinetics makes it questionable to correct M_bw _for the steady state insulin level in plasma to yield the M_bw_/I ratio, although this is often done. The high concentration of insulin at the effect site at the end of a glucose clamp probably changes *CL *very little for a large increment in plasma insulin. Correcting M_bw _for steady state plasma insulin also resulted in poorer correlations vis-à-vis the IVGTT.

Likewise, one may question whether baseline insulin should be subtracted from AUC_ins _when estimating M_bw _from an IVGTT test. Although being a logical and commonly used correction, disregarding the baseline strengthened the correlations in the present study. Inhibition of the endogenous glucose production taking place early during the IVGTT is likely to make the insulin concentration below baseline govern the disposition of both the exogenous and the endogenous glucose later during the test. Differences in the mathematical correlations between the glucose clamp and the IVGTT were fairly small, however, and we therefore conclude that correcting for baseline insulin can be done, but is not essential.

### Comparison with other methods

The precision by which our 12-sample IVGTT could predict insulin sensitivity stands out favourably in comparison with other and more complex approaches, as presented in a review by Borai *et al*. [1].

A previous study of MINMOD based on a series of 25 blood samples showed a linearity to the glucose clamp that was quite similar to the r^2 ^= 0.34 found here [13]. The new algorithms thus offered far better linearity than MINMOD in the present setting. MINMOD contains four unknown parameters that become gradually more difficult to estimate with good precision the fewer samples there are available. Moreover, MINMOD is not well suited for short sampling times. In contrast, the new algorithms included least-square regression estimation of only two parameters, *CL *and *V*_d_, which makes them less sensitive for a reduction of sampling time and/or sampling intensity. With 12 samples, *CL *and *V*_d _were estimated with the standard errors that averaged less than 10% (data not shown).

Tura *et al*. [11] recently compared the ratio of the glucose disappearance rate and AUC_ins _with *S*_I _and M_bw _in a retrospective analysis of studies comprising both volunteers and diabetic and postoperative patients who had undergone a frequently sampled 50-min IVGTT and a conventional 2-hour glucose clamp. Good correlations between these indices of insulin sensitivity were claimed for all subgroups. The basic equation used is quite similar to the one we propose on the top of Table 3. However, they did not use the ^10^log of AUC_ins _and corrected this area for the group average *S*_I _value. They also divided the expression by the sampling time, which we find questionable since plasma insulin but not *K*_G _decreases with time. This fact must be handled by using a unique equation for each sampling time, as in Tables 2 and 3.

### Limitations during surgery

The present study suggests two key algorithms, together with various modifications thereof, that may be used to estimate insulin sensitivity based on data derived from a short IVGTT performed in healthy volunteers. In a subsequent study, these algorithms will be validated in the pre- and postoperative settings. Our interest in this topic stems from a wish to study insulin resistance during surgery. Virtually all non-diabetic patients develop transient type 2 diabetes as a part of the stress response to surgery [4,5]. Too little research has been performed to investigate the reasons and consequences of this insulin resistance, which is probably due to the demanding and complex nature of both the glucose clamp and the IVGTT. In this setting, it is important that the blood sampling and the time and resources required for the test are kept low. Moreover, the test should impose only a slight burden on the body's physiology.

## Conclusion

The ratio of the slope of the glucose elimination curve and the AUC for plasma insulin during a short IVGTT showed a strong linear correlation (r^2 ^= 0.63-0.83) with the insulin sensitivity as obtained by the glucose clamp technique in healthy volunteers.

## Abbreviations

AUC: area under the curve; *CL*: clearance; IVGTT: intravenous glucose tolerance test; MINMOD: minimal model analysis; *V*_d_: volume of distribution; T_1/2_: half-life.

## Competing interests

The authors declare that they have no competing interests.

## Authors' contributions

RH provided the study idea, made the calculations, and wrote the manuscript. SL and FL assisted during the experiments. TN wrote the ethics application and arranged for the experiments.
